# MRGBP, a member of the NuA4 complex, inhibits DNA double‐strand break repair

**DOI:** 10.1002/2211-5463.13071

**Published:** 2021-02-20

**Authors:** Sabrina Rivero, Guillermo Rodríguez‐Real, Inés Marín, Pablo Huertas

**Affiliations:** ^1^ Department of Normal and Pathological Histology and Cytology University of Seville School of Medicine Spain; ^2^ Centro Andaluz de Biología Molecular y Medicina Regenerativa‐CABIMER Universidad de Sevilla‐CSIC‐Universidad Pablo de Olavide Spain; ^3^ Department of Genetics University of Seville Spain; ^4^ Present address: Institute for Research in Biomedicine (IRB Barcelona) Barcelona Institute of Science and Technology (BIST) Spain

**Keywords:** DNA repair, DNA‐end resection, MRGBP, recombination, TIP60

## Abstract

The repair of DNA breaks takes place in the context of chromatin and thus involves the activity of chromatin remodelers. The nucleosome acetyltransferase of H4 (NuA4) remodeler complex enables DNA break repair by relaxing flanking chromatin. Here, we show that MRG domain binding protein (MRGBP), a member of this complex, acts as a general inhibitor of DNA double‐strand break repair. Upon its downregulation, repair is generally increased. This is particularly evident for the stimulation of early events of homologous recombination. Thus, MRGBP has an opposing role to the main catalytic subunits of the NuA4 complex. Our data suggest that MRGBP acts by limiting the activity of this complex in DNA repair, specifically by narrowing the extent of DNA‐end resection.

## Graphical abstract

DNA repair takes place in the context of chromatin; thus, chromatin structure affects genome stability. In this article, we show that MRG domain binding protein, a member of the nucleosome acetyltransferase of H4 chromatin remodeling complex, acts as a general inhibitor of DNA double‐strand break repair, mostly inhibiting DNA end resection and homologous recombination, in stark contrast with other members of the same complex.

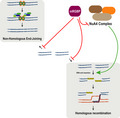

**Abbreviations**
53BP1: p53 binding protein 1
BRCA1: breast cancer 1
CtIP: CtBP interacting protein
DMEM: Dulbecco's modified Eagle's medium
DSB: DNA double‐strand break
HR: homologous recombination
IR: ionizing radiation
MDC1: mediator of DNA damage checkpoint 1
MRGBP: MRG domain binding protein
NHEJ: nonhomologous end‐joining
NuA4: nucleosome acetyltransferase of H4
PLA: proximity ligation assay
RPA: replication protein A
SMART: single molecule analysis of resection tracks
ssDNA: single‐ stranded DNA
TIP60: tat interacting protein, 60kDa

The stability of the genomic material is essential for cell and organismal fitness and survival. Thus, it is not surprising that a plethora of repair mechanisms have appeared in evolution to deal with chemical or physical alterations of the DNA. Among the possible DNA lesions, the breakage of the molecule, the so‐called DNA double‐strand break (DSBs), is known to be the most challenging to be repaired. Indeed, such DNA lesions can be repaired by several different mechanisms, including the error‐prone nonhomologous end‐joining (NHEJ) pathway and the error‐free homologous recombination (HR) pathway [1, 2]. Whereas NHEJ requires no homology and very little processing of the DNA ends, HR needs long tails of single‐stranded DNA (ssDNA) that are used to invade a homologous template during repair [1, 2]. Thus, it is the formation of this ssDNA, the so‐called DNA‐end resection, what effectively controls the choice between each DSB repair pathway. Mechanistically, resection is achieved by the combined action of several nucleases that degrade one strand at each side of the break in a 5′–3′ polarity [3]. But more importantly, a complex regulatory network controls if and when DNA resection machinery is activated [4, 5]. This requires the integration of multiple cellular signals and, mainly, the activation of the core resection factor CtBP Interacting protein (CtIP) [6]. Many different proteins are known to be involved in this network, including among many others the antagonistic roles of the pro‐resection factor breast cancer 1 (BRCA1) and the anti‐resection protein p53 Binding Protein 1 (53BP1) [7].

Additionally to the repair machinery itself, the efficient detection and repair of DSBs require the reorganization of the chromatin before and after repair takes place [8]. Indeed, different remodeling factors and histone‐modifying enzymes are involved in creating open chromatin structures at DSBs sites that are permissive for repair. This is the case of the highly conserved nucleosome acetyltransferase of H4 (NuA4) complex, that is specifically recruited to DSBs where it mediates the transition from compacted to open, relaxed chromatin to permit an adequate DSB repair [9, 10, 11, 12]. This role requires the action of different activities within the complex, catalyzed by the ATPase p400 and the histone acetyltransferase tat interacting protein, 60 kDa (TIP60) [9]. First, the p400 ATPase remodels the nucleosome organization by rapidly substituting the histone H2A for the H2A.Z variant [13]. This dynamic exchange is required for the creation of open chromatin domains at DSBs and for the subsequent acetylation of histone H4 by TIP60 [13, 14] that precedes the recruitment of several DNA repair proteins [15]. Moreover, TIP60 has also a direct role in damage signaling promoting ATM activation and the subsequent H2AX phosphorylation [16, 17] and has been proposed to be a key regulator of DSB repair pathway choice, favoring HR over NHEJ due to its capacity of inhibiting the local recruitment of 53BP1 [18]. In agreement, depletion of different human NuA4 subunits, including p400 or transformation/transcription domain associated protein, impairs the recruitment of HR proteins, including BRCA1 and the recombinase Rad51 [homologue to yeast Radiation sensitive 51 (RAD51)] [13, 15, 19]. The function of other hNuA4 subunits in this context is, however, less known. One of the less characterized members of the complex is MRG domain binding protein (MRGBP) [20]. This protein was recently identified in a genomewide screening for factors that regulate the balance between HR and NHEJ [21]. Strikingly, and contrary to the expectations for a NuA4 member, MRGBP depletion shifted the balance between HR and NHEJ toward the former.

Here, we show that MRGBP acts as a general inhibitor of DNA repair. Indeed, both NHEJ and HR seem to be repressed by this factor. However, the effect is particularly strong for HR. Specifically, MRGBP presence limits DNA‐end resection and, therefore, HR.

## Material and methods

### Cell lines and growth conditions

U2OS cells were grown in Dulbecco's modified Eagle's medium (DMEM; Sigma‐Aldrich, St Louis, MO, USA) supplemented with 10% FBS (Sigma‐Aldrich), 2 mm l‐glutamine (Sigma‐Aldrich), and 100 units·mL^−1^ penicillin and 100 μg·mL^−1^ streptomycin (Sigma‐Aldrich). U2OS cells bearing a copy of the DR‐GFP, SA‐GFP, or EJ5‐GFP reporter systems were grown in standard DMEM described above supplemented with 1 μg·mL^−1^ puromycin (Sigma‐Aldrich).

### siRNA transfection

siRNA duplexes were obtained from Sigma‐Aldrich or Dharmacon (Lafayette, CO, USA; Table S1) and were transfected using RNAiMax Lipofectamine Reagent Mix (Life Technologies, Carlsbad, CA, USA), according to the manufacturer's instructions.

### HR and NHEJ analysis

U2OS cells bearing a single copy integration of the reporters DR‐GFP (Gene conversion), SA‐GFP (SSA), or EJ5‐GFP (NHEJ) [22, 23] were used to analyze the different DSB repair pathways as previously described [24]. Four different parameters were considered: side scatter (SSC), forward scatter (FSC), blue fluorescence (407 nm violet laser BP, Filter 450/40), and green fluorescence (488 nm blue laser BP Filter 530/30). Finally, the number of green cells from at least 10 000 events positives for blue fluorescence (infected with the I‐SceI–BFP construct) was scored, considering the background of green fluorescence obtained in the samples without transduction with lentivirus harboring pBFP‐I‐SceI plasmid as previously described [23, 25, 26]. To facilitate the comparison between experiments, this ratio was normalized with siRNA control. At least three completely independent experiments were carried out for each condition and the average and standard deviation is represented.

### SDS/PAGE, western blot analysis, and immunoprecipitation

As described previously [24], protein extracts were prepared in 2× Laemmli buffer (4% SDS, 20% glycerol, 125 mm Tris/HCl, pH 6.8) and passed 10 times through a 0.5‐mm needle‐mounted syringe to reduce viscosity. Proteins were resolved by SDS/PAGE and transferred to low fluorescence PVDF membranes (Immobilon‐FL; Millipore, Billerica, MA, USA). Membranes were blocked with Odyssey blocking buffer (LI‐COR, Lincoln, NE, USA) and blotted with the appropriate primary antibody (Table S2) and infrared dyed secondary antibodies (LI‐COR; Table S3). Antibodies were prepared in blocking buffer supplemented with 0.1% Tween‐20. Membranes were air‐dried in the dark and scanned in an Odyssey Infrared Imaging System (LI‐COR), and images were analyzed with ImageStudio software (LI‐COR). Co‐immunoprecipitation experiments were performed as previously described [27] with the appropriate antibody (Table S2). Rabbit or mouse purified IgG (Sigma‐Aldrich) was used as a control.

### Immunofluorescence and microscopy

Those experiments were performed as previously described [24]. For replication protein A (RPA) foci visualization, U2OS cells knocked down for different proteins were seeded on coverslips. One hour after irradiation (10 Gy), coverslips were washed once with PBS followed by treatment with pre‐extraction buffer (25 mm Tris/HCl, pH 7.5, 50 mm NaCl, 1 mm EDTA, 3 mm MgCl_2_, 300 mm sucrose, and 0.2% Triton X‐100) for 5 min on ice. Cells were fixed with 4% paraformaldehyde (w/v) in PBS for 15 min. Following two washes with PBS, cells were blocked for 1 h with 5% FBS in PBS, co‐stained with the appropriate primary antibodies (Table S2) in blocking solution overnight at 4 ºC or for 2 h at room temperature, washed again with PBS, and then co‐immunostained with the appropriate secondary antibodies for 1 h (Table S3) in blocking buffer. After washing with PBS and dried with ethanol 70% and 100% washes, coverslips were mounted into glass slides using Vectashield mounting medium with DAPI (Vector Laboratories, Burlingame, CA, USA). RPA foci immunofluorescences were analyzed using a Leica Fluorescence microscope.

For 53BP1 visualization, U2OS cells were seeded and transfected as previously described. Once collected, cells were fixed with methanol (VWR, Radnor, PA, USA) for 10 min on ice, followed by treatment with acetone (Sigma) for 30 s on ice. Then, samples were immunostained as described above with the appropriate primary (Table S2) and secondary antibodies (Table S3). Images obtained with a Leica Fluorescence microscope were then analyzed using Metamorph to count the number, intensity, and size of the foci.

### SMART (single‐molecule analysis of resection tracks)

Single molecule analysis of resection tracks (SMART) was performed as described [28]. Briefly, cells were grown in the presence of 10 μm BrdU for < 24 h. Cultures were then irradiated (10 Gy) and harvested after 1 h. Cells were embedded in low‐melting agarose (Bio‐Rad, Hercules, CA, USA), followed by DNA extraction. DNA fibers were stretched on silanized coverslips, and immunofluorescence was carried out to detect BrdU (Table S2). Samples were observed with a Nikon NI‐E microscope, and images were taken and processed with the nis elements Nikon Software (Tokyo, Japan). For each experiment, at least 200 DNA fibers were analyzed, and the length of the fibers was measured with adobe photoshop cs4 (San Jose, CA, USA).

### Cell cycle analysis

We used the same protocol previously described in [24]. Briefly, cells were fixed with cold 70% ethanol overnight, incubated with 250 μg·mL^−1^ RNase A (Sigma) and 10 μg·mL^−1^ propidium iodide (Fluka, Buchs, Switzerland) at 37 ºC for 30 min, and analyzed with a FACSCalibur (BD, Franklin Lakes, NJ, USA). Cell cycle distribution data were further analyzed using modfit lt 3.0 software (Verity Software House Inc, Topsham, ME, USA).

### Proximity ligation assay

Proximity ligation assays (PLA) were performed using the Duolink PLA Kit (Olink Bioscience, Uppsala, Sweden) according to the manufacturer's protocol. Briefly, U2OS GFP‐mediator of DNA damage checkpoint 1 (MDC1) harboring cells were treated with ionizing radiation (IR; 10 Gy), incubated 1 h, and then collected. Coverslips were washed with PBS, fixed using PFA diluted in 4% PBS for 15 min at room temperature, and treated with triton 0.1% in PBS for 15 min. Then, cells were washed three times with PBS and blocked with Blocking Solution from Duolink PLA kit for 1 h at 37 ºC. Samples were incubated with primary antibodies against MRGBP and TIP60 (Table S2) overnight at 4 ºC, followed by MINUS and PLUS secondary PLA probes for 1 h at 37 ºC. Detection was carried out with the Duolink Detection Kit Red (Olink Bioscience). Cells were analyzed using a Leica fluorescence microscope.

### Statistical analysis

Statistical significance was determined with a Student's *t*‐test or ANOVA as indicated using prism software (GraphPad Software Inc., San Diego, CA, USA). Statistically significant differences were labeled with one, two, or three asterisks if *P* < 0.05, *P* < 0.01, or *P* < 0.001, respectively.

## Results

### MRGBP is involved in DNA DSB repair

As mentioned, previously we performed a genomewide study in human cells to identify candidate genes involved in the regulation of DSB repair pathway choice [21]. Among others, we found that MRGBP, a member of the NuA4 complex, seemed to favor NHEJ over HR [21]. Indeed, downregulation of MRGBP skewed the balance of DSB repair toward an increase in recombination [21]. So, we decided to study in detail the possible role of this protein in this essential process.

**Fig. 1:**
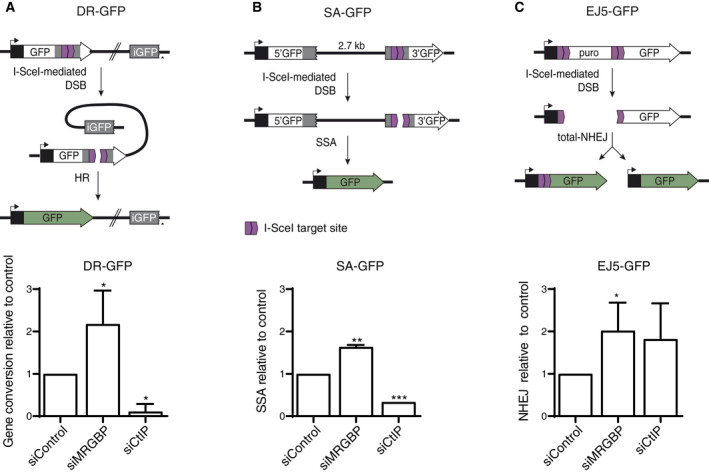
MRGBP depletion leads to hyper‐recombination. (A) Classical HR was measured as described in the methods section using the DR‐GFP reporter. An active GFP gene is formed upon gene conversion of an I‐SceI‐induced DSB (top). The percentage of GFP‐positive cells in U2OS cells bearing the reporter and depleted for the indicated proteins was scored and normalized as described in the methods section. The experiment was performed three times and the average and SD are plotted (bottom). Statistical significance was calculated using a Student*t*‐test comparing each condition to siControl cells. The asterisk represents *P* < 0.05. (B) Same as (A), but using the SSA reporter SA‐GFP. (C) Same as (A), but using the NHEJ reporter EJ5‐GFP.

Thus, first, in order to validate the genomewide results, we analyzed the impact of MRGBP depletion in specific DNA repair pathways using previously described, GFP‐based, DSB repair assays [22, 23]. We used the depletion of the bona fide DNA resection factor CtIP as a positive control, as it is known to increase NHEJ and decrease all types of HR [29, 30]. MRGBP and CtIP depletion is documented in Fig. S1A. In agreement with our published results, MRGBP depletion caused a general upregulation of HR, both the RAD51‐dependent gene conversion and the RAD51‐independent single‐strand annealing (Fig. 1A,B). Surprisingly, NHEJ was also upregulated after MRGBP downregulation (Fig. 1C), suggesting that MRGBP acts as a general suppressor of any type of DSB repair. As position in the cell cycle plays a crucial role in the DNA repair pathway choice, we checked that our results were not caused by a change in cell cycle distribution upon MRGBP depletion (Fig. 1B).

### MRGBP depletion leads to an increased recruitment of DNA repair proteins

**Fig. 2:**
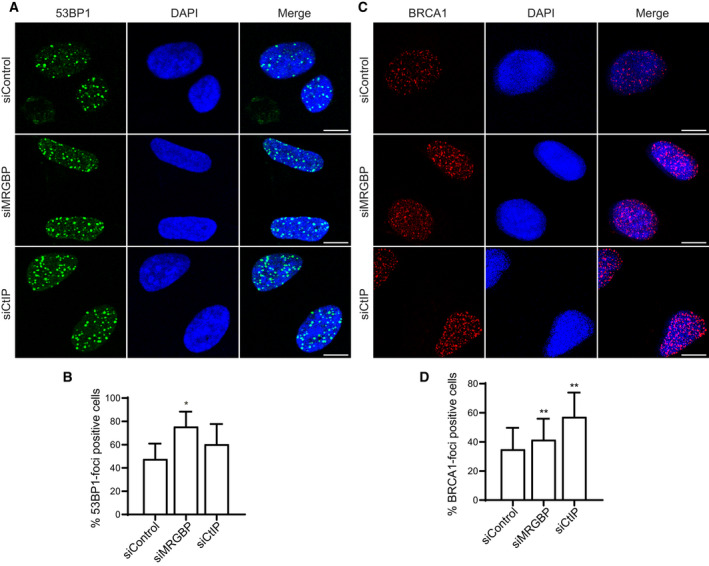
MRGBP depletion leads to increased recruitment of DNA repair proteins. (A, B) The formation of 53BP1 foci was tested in U2OS cells transfected with the indicated siRNAs 1 h after irradiation (10 Gy). The average and SD of three independent experiments are plotted (B) and representative images are shown (A). CtIP depletion was used as a positive control. Statistical significance was calculated using a paired Student *t*‐test comparing each condition to siControl cells. One or two asterisks represent *P* < 0.05 and *P* < 0.01, respectively. Scale bars represent 10 µm. (C, D) Same as (A) but for BRCA1 foci formation.

We then wondered if this increases in both NHEJ and HR rates correlated with a higher recruitment of different repair factors to sites of DNA damage. Thus, we analyzed the local recruitment of 53BP1, commonly associated with NHEJ [7, 31], and BRCA1, related to HR [7, 31], after MRGBP downregulation. The phosphorylation of histone H2AX (γH2AX) was used as a marker of DSBs. Again, depletion of CtIP was used as a positive control of altered DNA repair and foci formation. One hour after irradiation, MRGBP‐depleted cells exhibited a marked increase in 53BP1 foci, in line with the rise in NHEJ repair observed with the NHEJ reporter system (Fig. 2A,B). Similarly, accumulation of BRCA1 was also mildly but statistically significantly increased in MRGBP‐deficient cells, in agreement with the hyper‐recombination phenotype observed in SA‐GFP and DR‐GFP HR reporters (Fig. 2C,D). No changes were observed in nonirradiated samples (data not shown), arguing that such increase was not due to accumulation of endogenously created DNA damage.

### MRGBP inhibits resection

**Fig. 3:**
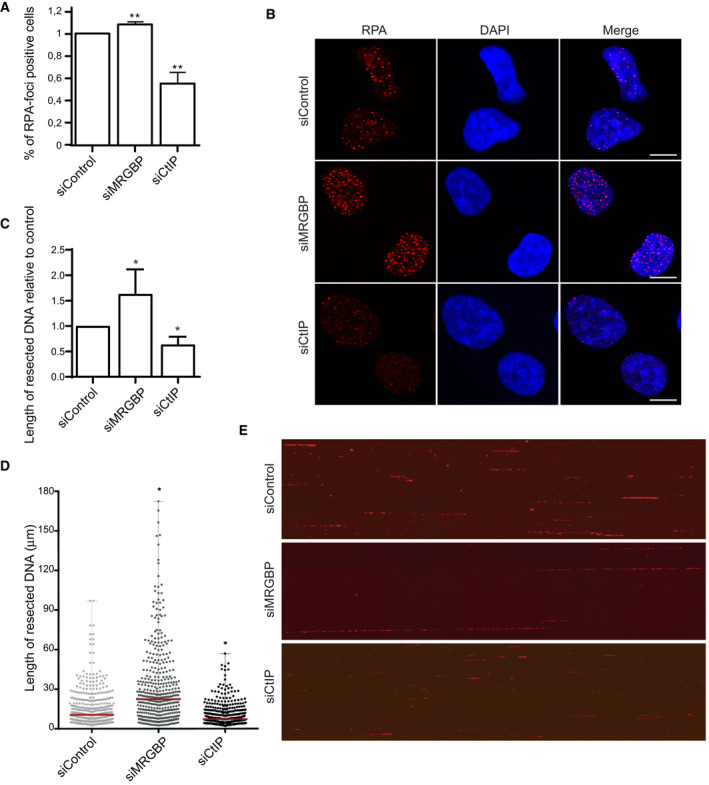
MRGBP inhibits resection. (A, B) DNA‐end resection was scored by the accumulation of RPA foci 1 h after irradiation (10 Gy) in U2OS cells transfected with the indicated siRNAs. The average and SD of four independent experiments are plotted (A) and representative images are shown (B). Statistical significance was calculated using a one‐way ANOVA test comparing each condition to siControl cells. Scale bars represent 10 µm. (C–E) Resection length on individual DNA fibers was calculated using SMART. At least 200 fibers were scored per condition. Resection length was normalized to the control sample. The average and SD of four independent experiments are plotted (C). The quantification of a representative experiment is shown in D and representative images are shown (E). Each point corresponds to the length of a single DNA fiber. The median is shown in red.

As mentioned, the balance between NHEJ and HR is maintained mainly at the level of DNA‐end resection initiation. Thus, it is not surprising that this process is heavily controlled by multiple layers of regulation. Among them, the interplay between 53BP1 and BRCA1, that show antagonistic roles, is one of the best defined [7]. Considering that the recruitment of both proteins is enhanced upon MRGBP depletion, we wondered if resection was inhibited, increased, or maintained to similar levels on such conditions. First, we checked the formation of RPA foci in cells exposed to DNA damage. As shown in Fig. S3, panels A and B, downregulation of MRGBP slightly but significantly increased the number of cells that were positive for RPA foci 1 h after exposing them to IR, in stark contrast with the effect of the depletion of the canonical resection factor CtIP. This result was confirmed using a second, independent siRNA targeting MRGBP (Fig. S2). The minimal increase we observed in RPA‐positive cells agreed with a role as an inhibitor of resection and explained why this factor appeared in our screening as favoring NHEJ [21]. So, albeit both NHEJ and HR are enhanced upon its downregulation, it seems that also the balance between both pathways is mildly skewed to favor NHEJ. To further resolve the changes in resection upon MRGBP depletion, we used SMART, a high‐resolution method to study DNA‐end resection in single DNA fibers [32]. Remarkably, downregulation of MRGBP did increase the median length of resected DNA (Fig. 3C). Moreover, we could also observe unusually long ssDNA fibers (Fig. 3D,E) in agreement with a hyper‐resection phenotype. These results support the implication of MRGBP in hampering all types of DNA repair, but with a greater effect on limiting the extent of DNA‐end resection.

### MRGBP antagonizes TIP60 and p400 in homologous recombination

**Fig. 4:**
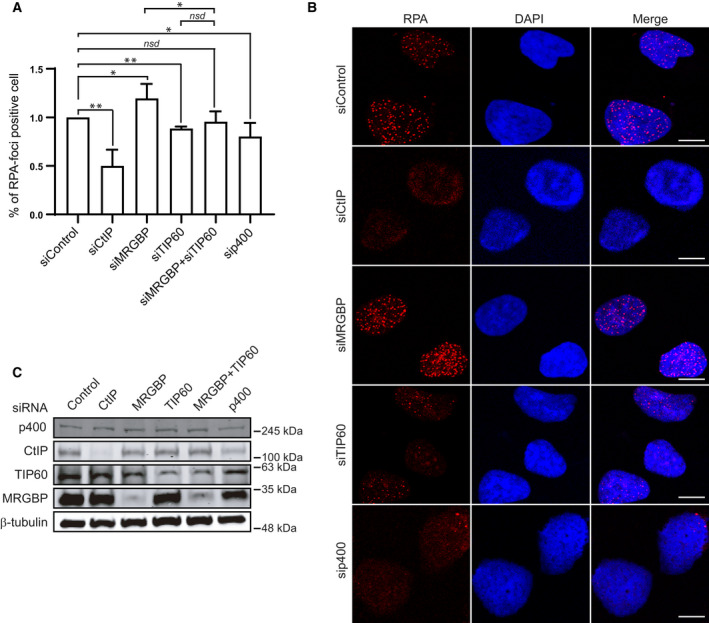
MRGBP antagonizes TIP60 and p400 in HR. (A, B) RPA foci formation was tested in U2OS cells transfected with the indicated siRNAs 1 h after irradiation (10 Gy). To facilitate the comparison, data were normalized to control cells. The average and SD of four independent experiments are plotted (A), and representative images are shown (B). Statistical significance was calculated using a one‐way ANOVA test. Comparisons between different pairs of data are shown, as indicated. One or two asterisks represent p < 0.05 and *P* < 0.01, respectively. Scale bars represent 10 µm. (C) Depletion was measured 72 h after siRNA transfection by western blot with the indicated antibodies. α‐Tubulin was used as a loading control.

MRGBP has been described as a component of the NuA4 complex, which strikingly has been shown to play the opposite role and to be required for the repair of DSBs by HR [15, 19, 33, 34, 35]. Thus, we decided to establish the genetic relationship for DNA‐end resection between MRGBP and the acetyltransferase TIP60 and the chromatin remodeler p400 ATPase, the two main subunits of the complex. As expected, and in agreement with the requirement of NuA4 in recombination, both TIP60 and p400 depletion slightly decreased the number of RPA‐positive cells after irradiation (Fig. 4). As previously observed, MRGBP downregulation had the opposite effect (Fig. 4). Importantly, depletion of TIP60 was epistatic over MRGBP downregulation, as the co‐depletion of both showed a phenotype similar to TIP60 simple depletion. Thus, our data indicated that MRGBP acted in the same genetic pathway as TIP60, likely by inhibiting its stimulatory role on resection.

### MRGBP interacts with NuA4 complex independently of DNA damage

**Fig. 5:**
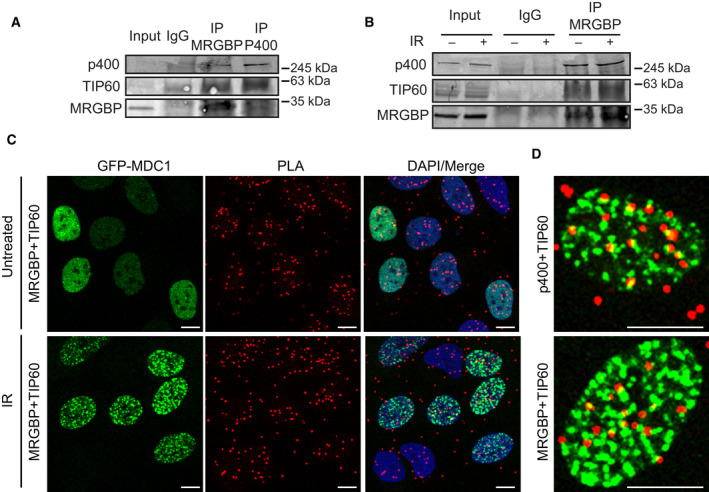
MRGBP interacts with NuA4 complex independently of DNA damage. (A) Whole‐cell extracts from U2OS cells were subjected to immunoprecipitation with anti‐MRGBP, anti‐p400, or control (IgG) antibodies. Immunoprecipitated proteins and 3% of the input were analyzed by western blot for the indicated proteins. (B) Whole‐cell extracts from control U2OS cells before (−) and 1 h after irradiation (+; 10 Gy) were subjected to immunoprecipitation with anti‐MRGBP or control (IgG) antibodies. Immunoprecipitated proteins and 3% of the input were analyzed by western blot for the indicated proteins. C PLA foci using MRGBP and TIP60 antibodies in cells expressing GFP‐MDC1 1 h after irradiation (10 Gy). Scale bars represent 10 µm. (D) A representative cell for (C) is shown at the bottom. PLA foci using TIP60 and p400 antibodies were used as a positive control (top).

One possibility to explain this opposite role of different components of the same complex was that MRGBP might be sequestering the NuA4 complex that would be released in a controlled manner in response to DNA damage. To test this hypothesis, we analyzed the composition of the complex in U2OS cells before and after irradiation. However, and as shown in Fig. 5A,B, MRGBP interacted with both TIP60 and p400 similarly both in the presence and absence of DBSs. To establish the cellular localization of such interaction, we used a PLA in irradiated cells expressing GFP‐MDC1, which is recruited to damaged DNA (Fig. 5C,D; controls for the specificity of the technique are shown in Fig. S3). Interestingly, despite the fact that both proteins continued interacting upon DNA damage appearance, they rarely do so on damaged chromatin, as revealed by co‐localization with MDC1 foci (Fig. 5D). The interaction between p400 and TIP60 was monitored as a positive control (Fig. 5D), showing a similar pattern.

## Discussion

Here, we show that MRGBP, a poorly studied member of the NuA4 complex, seemed to reduce TIP60 pro‐resection function. MRGBP depletion increases resection extension causing hyper‐resection, in a manner that is completely dependent on the presence of TIP60. Strikingly, such depletion favors the quantity of DSB repair by both HR and NHEJ, what is dependent on a hyper‐accumulation of repair factors involved in either pathway. We could wonder why such protein has appeared in evolution and what could be the benefit of a general limitation of repair. Our hypothesis is that the efficiency of the repair must be governed mostly by the quality rather than by the quantity of the process. Indeed, MRGBP presence might limit the activity of the NuA4 complex at sites of DSBs, favoring a chromatin environment in which repair can happen at a controlled pace. In this scenario, the absence of MRGBP would deregulate TIP60 activity, creating a chromatin landscape that facilitates the accumulation of repair factors. This unchecked repair might be faster, therefore increasing the number of breaks sealed at a given time, but probably less effective and most likely will result in an increased genomic instability due to a reduction of the quality of the repair. Indeed, the role of the NuA4 complex in facilitating repair, mainly by stimulating DNA‐end resection and HR is well established [9, 10, 11, 12, 13, 17, 35]. So, its untamed and unlimited activity agrees with the hyper‐resection phenotype observed upon MRGBP depletion. While it might be possible that hyper‐resection does not generally block repair, it could render fundamental changes in the type of repair event that takes place. In fact, classical NHEJ will be mostly blocked by resection, but the exposure of short microhomologies during resection will hyper‐stimulate the micro‐homology‐mediated end joining, a highly mutagenic pathway. Regarding HR, that is completely dependent on DNA‐end processing, hyper‐resection can also shift the balance between the different subpathways. On the one hand, it will favor longer gene conversion tracks, as the extent of ssDNA will increase. On the other hand, it will also difficult the capture of the second DNA end required for the formation of a double Holiday Junction during classical recombination [36]. Finally, hyper‐resection will greatly facilitate the exposure of intrachromosomal repeats, favoring the single‐strand annealing homology‐mediated repair, thus increasing intrachromatid events and the deletion of big regions on the DNA [1]. Therefore, we speculate with the idea that MRGBP is responsible of taming the activity of the NuA4 complex, favoring slower but more accurate repair pathways, thus increasing the stability of the genome.

## Conflict of interest

The authors declare no conflict of interest.

## Author contributions

SR IM, and GR‐R shared the experimental work. PH supervised the work.

## Supporting information


**Fig. S1.** MRGBP depletion does not affect cell cycle distribution.

**Fig. S2.** Depletion of MRGBP with a different siRNA inhibits resection.

**Fig. S3.** Controls for PLA technique.

**Table S1**. siRNAs used.
**Table S2**. Primary antibodies used.
**Table S3.** Secondary antibodies used.

## Data Availability

All data generated or analysed during this study are included in this published article (and its supplementary information files).
