# Supplementary material for: MRGBP, a member of the NuA4 complex, inhibits DNA double‐strand break repair
Source: FEBS Open Bio. 2021 Feb 20;11(3):622–32. doi: 10.1002/2211-5463.13071 (PMC7931222; doi:10.1002/2211-5463.13071)
Supplement: Supplementary file 4 — Table S1. siRNAs used. Table S2. Primary antibodies used. Table S3. Secondary antibodies used. [file FEB4-11-622-s004.docx]

**Table S1**. siRNAs used.

| Target gene | Description/Sequence | Source/Reference |
| --- | --- | --- |
| Non-targeting scramble siRNA (siControl) | ON-TARGETplus Non-targeting Pool | Dharmacon  (D-001910-10-20) |
| CtIP (siCtIP) | GCUAAAACAGGAACGAAUC | Sigma-Aldrich |
| MRGBP (siMRGBP) | ON-TARGETplus human MRGBP SMARTpool | Dharmacon  (SO-2547860G) |
| MRGBP (siMRGBP-2) | GCAAAGACAAAGAGAAGAA | Sigma-Aldrich |
| TIP60 (siTIP60) | CCUCAAUCUCAUCAACUAC | Sigma-Aldrich |
| P400 (siP400) | UGAAGAAGGUUCCCAAGAA | Sigma-Aldrich |

**Table S2**. Primary antibodies used. WB, western blotting; IF, immunofluorescence; SMART, Single Molecule Analysis of Resection Tracks; IP, immunoprecipitation.

| Target protein | Source | Reference | Application |
| --- | --- | --- | --- |
| γ-H2AX | Mouse | Millipore (05-636) | IF |
| γ-H2AX | Rabbit | Cell Signaling (2577L) | IF |
| 53BP1 | Rabbit | Novus Biologicals (NB100-304) | IF |
| BRCA1 | Mouse | Santa Cruz (sc-6954) | IF |
| RPA32 | Mouse | Abcam (ab2175) | IF |
| BrdU | Mouse | Amersham (RPN202) | SMART |
| MRGBP | Rabbit | Sigma-Aldrich (HPA-0127-012) | WB, IP |
| CtIP | Mouse | R. Baer (14.1) | WB |
| α-tubulin | Mouse | Sigma-Aldrich (T9026) | WB |
| β-actin | Rabbit | Abcam (ab8227) | WB |
| TIP60 | Mouse | Santa Cruz (sc-166323) | WB |
| P400 | Rabbit | Abcam (ab70301) | WB, IP |

**Table S3**. Secondary antibodies used. WB, western blotting; IF, immunofluorescence; SMART, Single Molecule Analysis of Resection Tracks.

| Target protein | Reference | Application |
| --- | --- | --- |
| IRDye 680 goat anti-mouse IgG (H+L) | LI-COR (929-68070) | WB |
| IRDye 680 goat anti-rabbit IgG (H+L) | LI-COR (929-32211) | WB |
| Alexa Fluor 594 goat anti-mouse (H+L) | Invitrogen (A11032) | IF, SMART |
| Alexa Fluor 488 goat anti-rabbit (H+L) | Invitrogen (A11034) | IF |

**Supplementary Figure 1. MRGBP depletion does not affect cell cycle distribution.**

**A**, A representative western blot analysis of CtIP, MRGBP and a-tubulin in U2OS cells after transfection with the indicated siRNAs. **B**, Cell cycle distribution after downregulation of the indicated genes with siRNAs. The resultant percentages of cells were plotted. The average and s.d. of three independent experiments are shown.

**Supplementary Figure 2. Depletion of MRGBP with a different siRNA inhibits resection.**

**A** U2OS cells were transfected with the indicated siRNAs and 72 hours after transfection, a total lysate was prepared and analysed by western blot for the indicated proteins. **B** RPA foci formation was tested in U2OS cells transfected with the indicated siRNAs 1 h after irradiation (10 Gy). In order to facilitate comparison, data was normalized to control siRNA. Data are the average of three independent experiments. Statistical significance was calculated using a One-way ANOVA test. Two asterisks represent p<0.01.

**Supplementary Figure 3. Controls for PLA technique.** PLA foci using only secondary

antibodies as a negative control in cells expressing GFP-MDC1 cells 1 h after irradiation

(10 Gy).
